# Falling on deaf ears: interpreters as cultural brokers in mental healthcare

**DOI:** 10.1192/bjb.2023.90

**Published:** 2024-04

**Authors:** Jordan Bamford, Seri Abraham, Mustafa Alachkar, Adeola Akinola

**Affiliations:** 1 University of Manchester, UK; 2 Pennine Care NHS Foundation Trust, UK

**Keywords:** Ethics, education and training, ethnicity, interpreting, language

## Abstract

Communication is the cornerstone of mental healthcare. In the UK, many people who will need access to mental health services do not possess English as their first language. In this editorial, we seek to examine current policy and guidance with respect to interpreting in mental healthcare, and explore the benefits and challenges of interpretation and the ethical implications to consider. We focus on how mental health services could better engage with interpreters as cultural brokers to understand cultural expressions of distress. We conclude by suggesting an education and research agenda which could decrease ethnic disparities in mental healthcare.

## Language barrier: the need for interpreters in mental healthcare

According to the 2011 census, 4.2 million people in the UK reported that English is not their first language,^1^ and many of these people will require access to mental health services. Within mental healthcare, language barriers may contribute to difficulty in assessment of mental health difficulties, limiting understanding of cultural expressions of distress, jeopardising the therapeutic relationship between clinician and patient, preventing disclosure of stigmatising information and hindering patient-centred communication in mental healthcare.^2,3^ Furthermore, navigating access to and engagement with mental healthcare services can be even more challenging if the patient does not speak English.^4^ Language barriers can contribute to misdiagnosis, empathic failure, poor working alliances and worse treatment outcomes.^5^ In the UK, experience of language discordance between patient and clinician disproportionately affects the care of people from minority backgrounds^6^ and vulnerable groups such as refugees and asylum seekers with increased risk of poor mental health outcomes.^7,8^ Language barriers can therefore limit the accessibility of physical and mental health services.^9^ As a result, some of those with the greatest need for mental health services have challenges accessing care. This in turn exacerbates the health inequalities that are commonly seen in mental health access and outcomes between different groups in society.

Notwithstanding the different meanings that the word ‘interpreting’ has in general, and in psychiatry in particular, we use the word and its derivatives to refer to the use of a professional (an interpreter) to carry out verbal translation in clinical settings from and into English when the patient's first language is not English. It has been evidenced that quality of care is reduced when patients with little English proficiency are not provided with interpreters.^5,10^ It is therefore imperative that a good-quality mental health service has the capacity to ensure interpreters are available when needed to overcome language barriers. Whereas language barriers can negatively influence access to and engagement with mental health services, the provision of interpreters can decrease the frequency of missed appointments, influence the effectiveness of consultations and have a positive impact on the patient's experience and health outcomes.^11^ Interpreters can operate as cultural brokers, wherein they provide clarification of expressions of distress and make a consultation more nuanced than literal word-for-word translation. This can help overcome sociocultural differences between the patient and the clinician, for example, providing insight into how a patient's behaviours or thoughts might be understood in their cultural context and whether that would be considered acceptable. However, current policy and guidance often fail to consider the specific challenges and opportunities for interpreters in mental healthcare.

## Current policy and guidance

From an overarching global perspective, equality of access to health services is highlighted in a variety of documents and declarations, including the European Convention for the Protection of Human Rights and Fundamental Freedoms, the United Nations Convention on the Rights of the Child, the Human Rights Act, the Equality Act and the National Health Service (NHS) constitution, to name a few.^12–14^ In the UK, the NHS has produced specific guidance for commissioners of healthcare in relation to the provision of interpreting and translation services. This guidance is underpinned by the principle that a patient should be able to access services in such a way that their language and communication requirements do not prevent them from receiving the same quality of care as others.^11^ General Medical Council (GMC) guidance indicates that the doctor must make all possible efforts to ensure effective communication with patients; this would include meeting the communication needs of a patient who does not speak English.^15^ Of relevance to mental healthcare, the GMC's guidance on decision-making and capacity explicitly states that a patient must be given information in a way which they can understand and retain; specifically, you should use an interpreter or translation service if the patient has difficulty with understanding spoken English.^16^ Similarly, the British Psychological Society has produced guidance which encourages the use of interpreters where needed, to ensure that access to and efficacy of therapy are not determined by English proficiency.^17^

Both NHS England and NHS Scotland have guidance which proposes that when there is a language barrier, a professional interpreter should be offered, and that doctors should avoid using family/relatives and children as interpreters.^11,18^ In 2017, the Office for Health Improvement and Disparities in the UK published guidance on language interpreting and translation in relation to migrant health, also indicating the profound need for ensuring that interpreters are provided when needed. The aforementioned policy also highlights how a person may possess good conversational fluency in English but not the proficiency to understand or discuss health-related information.^19^ Furthermore, this policy specifically indicates that the use of children for interpretation is never justifiable, that an interpreter should be present in any consultation involving child safety or gender-based violence, and that automated online translating systems (such as Google Translate) should generally be avoided.^19^

Generally, there is a lack of specific guidance relating to the challenges and opportunities of interpreting a mental health consultation. Specifically, no policy highlights the benefits of the interpreter as a cultural broker. Mediating between two different cultural backgrounds could allow for more accurate assessment, diagnosis and treatment, and for the patient it would bolster more trust, respect and satisfaction with services. Better policy and guidance on interpreting in mental healthcare could bolster the cultural competence of the system. It is important to consider the specific benefits of interpreting and balance this with some of the challenges.

## The impact of overcoming language barriers

Overcoming language barriers yields many benefits for clinicians, patients and the healthcare system, which is especially important at a time of low public satisfaction with the healthcare system.^20^ The Office for Health Improvement and Disparities indicates that working with professional interpreters ensures accuracy and impartiality, which in turn minimise the legal risk of misinterpretation of important clinical information and safeguarding risks.^19^ Thus, for example, interpreter-mediated consultations could contribute to identifying and supporting women experiencing gender-based violence. At present, members of ethnic minority groups whose first language is not English may use members of their family who are more proficient with English as interpreters. Although this may present a practical solution for the language barrier, it could be considered that the provision of a professional interpreter in this context would allow family members and friends to attend appointments and support the patient without the pressure of interpreting and help foster trust with the patient.^19^ Avoiding relying on family members to interpret may ensure no issues with breaching confidentiality or missing safeguarding issues.^21^ Evidence suggests that the use of interpreters ensures that patients without English engage better with services and treatment.^22^

In the context of the mental health consultation, interpreters can provide cultural explanations or advice and can be thus considered cultural brokers.^23–25^ This in turn promotes a holistic assessment of the patient, as meaning may be lost without cultural context. Interpreter provision can contribute to the aim of providing equal access to healthcare for refugees and immigrants in the UK.^25,26^ Furthermore, it may be considered that for the clinician's own practice, working with an interpreter allows for the understanding of different expressions of distress, bolstering understanding and relevance of cultural context and constructions, and improving knowledge of different explanatory health beliefs.^27^ At present, this is not a focus in interpreter-mediated consultations; in the clinical environment, there is often just a focus on literal word-by-word translation, with a loss of sociocultural context.

## Challenges of interpreting in mental health settings

The outlined benefits of interpreting are significant, but it is important to be mindful of the challenges associated with the use of interpreters. Some tentative evidence suggests that compared with language-concordant care, interpreter-mediated care may be associated with less patient participation, poorer relationship quality, less cultural understanding, reduced treatment engagement and worse levels of satisfaction for both patient and clinician.^28^

Having an interpreter present can change the dynamics at play in a consultation. In language-concordant consultations, there is the clinician and the patient, whereas in interpreter-mediated consultations, there is another party to consider, leading to three dyads: the clinician–interpreter dyad, who share a language and a position of power; the interpreter–patient dyad, who share a language and possibly a common culture; and the patient–clinician dyad. The interaction – and, at times, tension – between these dyads is key. Some research suggests patients may feel infantilised by the process of interpreting.^29^ Where an interpreter and clinician can work well together, they may foster a relationship with the patient in which the interpreter represents an extension of the clinician, as part of a ‘professional body’ interviewing the patient. However, this form of interaction is limited if the clinician and interpreter lack training in working together in mental health settings. One study in a Swiss hospital found that interpreters’ socioeconomic position was often closer to that of doctors/nurses than to that of patients, and that in some ways this meant they aligned themselves more with the healthcare staff, thereby exacerbating the power imbalance already existing between the clinician and the patient.^23,26^ Although this may not be the case in the UK, it may still be assumed by the patient, and it is therefore important to consider how such perceptions create difficulty for the development of trust and a therapeutic relationship between the clinician and the patient. Generally, therapists have reported that the process of exploring negative feelings can be made more difficult when working with an interpreter as a conduit.^30^ Developing a therapeutic relationship where interpreters are used can take time, and it may be beneficial to acknowledge that openly in a therapy session and for it to become a topic to think about together, especially in relational and exploratory therapies.^31^

In mental healthcare settings, the role of the therapeutic relationship and the building of trust and confidence are of great significance, and the interpreter plays a crucial part in building that relationship. Concerningly, interpreters often lack specific training for working in mental health services, and this can negatively affect the interaction between the clinician and patient. Presently, interpreters in the NHS must be registered with the National Register of Public Service Interpreters.^11^ This register has an associated code of conduct, which mentions the need to act impartially and avoid prejudice related to religion, race, politics, gender or age.^32^ However, there is no requirement for mandatory training on topics such as equality and diversity, and no specific need to have training in mental health consultations, nor is there any guidance for acting as a cultural broker to gain sociocultural context for the patient's thoughts and behaviours. This lack of standardisation may lead to differential quality of interpreting services for patients.

Jidong et al (2020) found that interpreters can struggle with the sensitive nature of a mental health consultation and may have difficulty ensuring accuracy. It can be very challenging to convey the meaning of expressions which are culture-bound in another language. Expressions of distress can vary significantly across cultures; they are influenced by how notions of the self and the mind are conceptualised in cultures. Differences in how individuals present with distress have contributed to underestimation of psychological distress among ethnic minorities, who, for example, may be more likely to describe psychological distress in terms of physical symptoms.^33,34^

Although we have discussed the perceived benefits of having an interpreter from the same culture as the patient's, we recognise how this may produce challenges too. It may potentially provoke a sense of shame and envy from the patient towards the interpreter. If the community shared by the patient and the interpreter is relatively small, they may have overlapping social networks, which raises issues with trust for the patient and can lead to a sense of stigma, deterring engagement with the clinician. An interpreter can be a source of fear or anxiety for certain groups, especially refugees who may have fled political persecution and remain worried about their own safety or that of their family or loved ones. Moreover, if the interpreter and patient are from a similar group (e.g. a refugee background) or if they share similar experiences and perspectives of suffering, this may create more challenges for the interpreter in remaining professional and neutral. For the interpreter, hearing similar experiences of suffering being repeated in front of them can contribute to vicarious traumatisation, for which interpreters describe very little organisational support.^35^

## Ethical considerations for the interpreted mental health consultation

There are many ethical considerations to be mindful of with respect to the use of interpreters in mental healthcare. Broadly, as language barriers can impede health service delivery, it can be considered that the failure to address these barriers can constitute malpractice and, where institutionalised, can be an ethical, civil or human rights violation.^26,36^ When interpreters are used, the duty of an interpreter in helping to overcome the obstacle of language discordance is in many ways an act of beneficence and non-maleficence. An more complex area is related to respecting the patient's choice of interpreter; this requires a careful balance of patient autonomy and non-maleficence. In a situation where a patient preferentially opts for a family member over a professional interpreter, there would be concerns with the use of a family member relating to confidentiality and impartiality as discussed previously. However, this must be balanced with respect for the patient's choice and a consideration of how interpretation through a family member could encourage the patient to open up during a consultation.

Examining interpreter standards globally, two of the main ethical issues identified are confidentiality and impartiality.^37^ For the interpreter, ensuring that the contents of the mental health consultation remain confidential is of utmost importance, so as to build trust with the patient,^38,39^ and this is highlighted in the code of practice for interpreters in the NHS.^32^ More nuanced for the mental health consultation are the issues of whether the interpreter has worked with the patient before and whether to disclose that information to the mental health professional, thereby risking a breach of the patient's confidentiality.^38^ In these circumstances, a careful balancing is required of the patient's right to privacy and consideration of possible consequences if information is not shared.

The topic of the interpreter's impartiality is an area which is both complex and controversial. Some authors have argued for the interpreter being impartial owing to concerns about poor-quality care due to the interpreter's lack of psychiatric training.^40^ Despite this aim of providing an unbiased exchange of communication, the reality is that interpreters do not simply transmit information; the evidence suggests that they are active participants in the diagnostic process and adapt to the needs of the patient.^41,42^ Interpreters should be mindful that when they communicate what the patient is saying, they should do this with the patient's level of actual capability and refrain from providing assistance,^43^ as this is key information for the clinician. It bears consideration that the presence of a friend or family member in an interpreted consultation (if the patient consents) could help resolve concern about impartiality.

## Considerations for the future

Presently, interpreters used in mental health settings often do not undergo specific training. We would argue that the needs and challenges associated with interpreting a mental health consultation necessitate specialist training. As we have discussed, the potential benefits of considering the interpreter as a cultural broker are significant. This is a underutilised resource which ultimately could improve ethnic minority disparity in mental healthcare. For medical training too, clinicians must have early opportunities in their careers for training and working with interpreters in mental health. A more collaborative approach to care between psychiatrists and interpreters will maximise the benefits of an interpreter-mediated consultation. Specific emphasis on and training in working with an interpreter as a cultural broker are needed. Much evidence has shown the demands and difficulties of the interpreter's role. We identify that there is a need for further support and debriefing for interpreters, given the challenges of these consultations.

From a research agenda perspective, there is a need for more research examining the barriers to accessing healthcare for those who do not speak English, as well as the deterrents to providing interpreters in healthcare settings. Qualitative research to examine the psychiatrists’ perspective in an interpreter-mediated clinical interview could inform future training needs, as would research which explores patients’ experiences of having a mental health consultation with an interpreter. This could provide a framework to consider what factors facilitate or hinder effective communication and the use of the interpreter as a cultural broker. We also identify that more research is needed to explore the perspective of interpreters, to fully capture their experience of the process of interpreting in mental health and examine how they are trained, supported and debriefed (especially when exposed to possibly traumatising experiences). Overall, we identify the need for exploration of the training received by interpreters used in mental health settings, to ensure a degree of standardisation.

## About the authors

**Jordan Bamford**, MB, BAO, MSc, is a National Institute for Health Research academic clinical fellow with the Division of Psychology and Mental Health, University of Manchester, UK. **Seri Abraham**, MBBS, MRCPsych, MSc, is a consultant psychiatrist with Pennine Care NHS Foundation Trust, UK, and honorary senior lecturer at Manchester Metropolitan University, UK. **Mustafa Alachkar**, MRCPsych, is a consultant psychiatrist and psychotherapist with Pennine Care NHS Foundation Trust, UK. **Adeola Akinola**, MBChB, MRCPsych, PGDip, LLM, FHEA, is a consultant psychiatrist with Pennine Care NHS Foundation Trust, UK, and a lecturer in ethics and law at the University of Manchester, UK.
